# Experience of parents in delivering pediatric *tuina* to children with symptoms of attention deficit hyperactivity disorder during the COVID-19 pandemic: qualitative findings from focus group interviews

**DOI:** 10.1186/s12906-023-03891-3

**Published:** 2023-02-18

**Authors:** Shu-Cheng Chen, Hui-Lin Cheng, Dong-Dong Wang, Shanshan Wang, Yue-Heng Yin, Lorna Kwai-Ping Suen, Wing-Fai Yeung

**Affiliations:** 1 School of Nursing, the Hong Kong Polytechnic University, HKSAR, China; 2 Yantai Aviation Medical Room, Shandong Airlines, Yantai, China; 3 School of Nursing, Tung Wah College, HKSAR, China

**Keywords:** Acupressure, Attention deficit disorder with hyperactivity, Massage, Medicine, Chinese traditional, Qualitative research

## Abstract

**Background:**

Evidence suggests that pediatric *tuina*, a modality of traditional Chinese medicine (TCM), might have beneficial effects on the symptoms of attention deficit hyperactivity disorder (ADHD), such as overall improvements in concentration, flexibility, mood, sleep quality, and social functioning. This study was conducted to understand the facilitators and barriers in the delivery of pediatric *tuina* by parents to children with ADHD symptoms.

**Methods:**

This is a focus group interview embedded in a pilot randomized controlled trial on parent-administered pediatric *tuina* for ADHD in preschool children. Purposive sampling was employed to invite 15 parents who attended our pediatric *tuina* training program to participate voluntarily in three focus group interviews. The interviews were audio-recorded and transcribed verbatim. The data were analyzed through template analysis.

**Results:**

Two themes were identified: (1) facilitators of intervention implementation and (2) barriers to intervention implementation. The theme of the facilitators of intervention implementation included the subthemes of (a) perceived benefits to children and parents, (b) acceptability to children and parents, (c) professional support, and (d) parental expectations of the long-term effects of the intervention. The theme of barriers to intervention implementation included the subthemes of (a) limited benefits for children’s inattention symptoms, (b) manipulation management difficulties, and (c) limitations of TCM pattern identification.

**Conclusion:**

Perceived beneficial effects on the children’s sleep quality and appetite and parent–child relationships, as well as timely and professional support, mainly facilitated the implementation of parent-administered pediatric *tuina*. Slow improvements in the children’s inattention symptoms and the possible inaccuracies of online diagnosis were the dominant barriers of the intervention. Parents have high expectations for the provision of long-term professional support during their practice of pediatric *tuina*. The intervention presented here can be feasibly used by parents.

## Background

Attention deficit hyperactivity disorder (ADHD) is one of the most prevalent neurodevelopmental conditions during childhood with average prevalence approximately 5% [1]. Its three core symptoms are inattention, hyperactivity, and impulsivity [2]. According to a 2016 national survey in the US, 64% of children with ADHD have one or more other mental, emotional, or behavioral disorders [3]. Psychiatric comorbidities in those with ADHD are varied and include learning disorders, sleep disorders, oppositional defiant disorders, anxiety disorders, intellectual disability, language disorders, mood disorders, and conduct disorders [4]. A longitudinal study with a 16-year follow-up on ADHD persistence in adulthood revealed that in 60% of children with ADHD, symptoms persist into adulthood [5]. Therefore, children must receive timely and cost-effective nonpharmacological interventions at a young age.

Conventional treatments for ADHD include medication and behavioral therapy [6, 7]. Even FDA-approved medications like methylphenidate, are frequently reported to have several potential side effects (e.g., dizziness, headaches, moodiness, and irritability) despite their immediate effects on the core symptoms of ADHD [8, 9]. Behavioral therapy is the first-line intervention for children under 6 years of age but requires a high level of family participation [10]. Pediatric *tuina,* also called pediatric *anmo* or traditional Chinese medicine (TCM) pediatric massage, is a special massage therapy for infants and children. Previous studies have attempted to explore the effects of pediatric *tuina* on numerous clinical conditions and diseases [11], such as diarrhea [12], anorexia [13], torticollis [14], cerebral palsy [15], scleroderma [16], constipation [17], infantile malnutrition [18], and fever [19] and in the promotion of the growth and development of healthy children in China [20]. *Pediatric tunia* is an external therapy involving the stimulation of specific areas or acupoints of the body through various manipulation techniques, such as pushing, kneading, pressing, rotating, nipping, circular, and pounding [20, 21]. Pediatric *tuina* produces various kind of stimuli on the skin, which could be sensed by surface sensory receptors and transferred to the central nervous system, inducing a series of protective adaptive homeostatic activities [22, 23]. A previous study also showed that in young children, the skin can rapidly regulate basic and adaptive homeostatic responses with a low compensatory basal level of stress-responsive enzymes, thus enabling a broad range of responses [24]. In a systematic review of 11 clinical studies on the use of massage in children with ADHD, seven works used pediatric *tuina* as the intervention. These studies reported that pediatric *tuina* might have beneficial effects on improving children’s concentration, flexibility, mood, sleep, social functioning, and overall condition [25].

In China, pediatric *tuina* has been increasingly developed as a parent-delivered intervention due to its simplicity and convenience [26]. Many experimental studies have demonstrated the multiple benefits of parent-administered behavioral interventions for ADHD [27–30] or the feasibility and effects of parent-administered massage-related interventions [31, 32]. However, only a few clinical trials have been conducted on pediatric *tuina* for ADHD with validated outcome measures to explore its specific and nonspecific effects. In addition, in-depth qualitative feedback regarding the administration of pediatric *tuina* from parents is lacking. Therefore, we recently conducted a pilot randomized controlled trial (RCT) on parent-delivered pediatric *tuina* in children with ADHD. Focus group interviews were also performed on the participants*.* The aim of this focus group study is to provide an in-depth understanding of the facilitators and barriers in intervention implementation and to explore the other beneficial effects of *tuina*.

## Methods

**Fig. 1:**
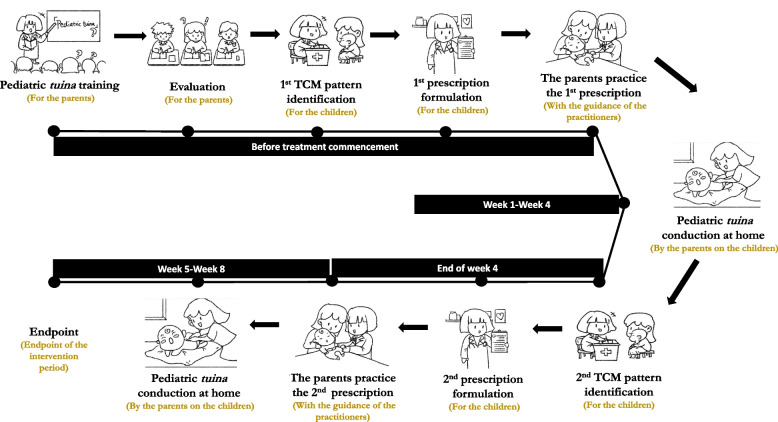
The procedure of parent-administered pediatric *tuina* intervention implementation

This project was registered in ClinicalTrials.gov (Identifier: NCT04237259). Ethical approval was obtained from Hong Kong Polytechnic University (HSEARS20190824002) and Shandong University of Traditional Chinese Medicine Affiliated Hospital ([2019] 伦审第 (044) 号–KY). This article reports on the qualitative component of a pilot RCT on parent-administered pediatric *tuina* for children with ADHD symptoms. In the RCT, parents were systematically trained on pediatric *tuina* intervention by TCM practitioners in five online training sessions (1 h for each training session) and delivered pediatric *tuina* to their children at home for 2 months. All study-related procedures were modified to online settings because of the COVID-19 pandemic. The details of the intervention and results have been described in other publications [33–35], and the procedure of the intervention implementation is shown in Fig. 1. The consolidated criteria for reporting qualitative research (COREQ) checklists were used to guide the reporting of this study [36].

### Aim

The aim of this study is to understand the facilitators and barriers in the delivery of pediatric *tuina* by parents to their children with ADHD symptoms.

### Design

This study employed a qualitative approach with the use of semi-structured focus group discussions. The research methodology allows for a great understanding of specific topics and active participation by participants, who can be expected to contribute their expertise and experience.

### Setting and participants

The focus group interviews were conducted online by using Tencent Meeting software, which is commonly used in business, municipal services, finance, education, healthcare, and other industrial fields and is widely used online and for interviews, learning, and training [37]. An online focus group interview was performed because it is a convenient and effective method for collecting the experience of participants from different geographic areas. The feasibility of online focus group interviews has been proven by many previous studies [38]. The setting of the study encompassed two organizations: Hong Kong Polytechnic University (School of Nursing) in Hong Kong and Shandong University of Traditional Chinese University Affiliated Hospital (Pediatric *Tuina* Center) in Jinan City.

Participants were parents with children with ADHD. They were recruited from the pilot RCT and by using purposive sampling. By applying homogeneous purposive sampling, the participants for the first two interview sessions were invited from the participants in the intervention group who were willing to attend the interview for a comprehensive understanding. For the third interview session, purposeful random sampling was used to minimize the potential bias due to pure criterion sampling [39]. The participants were invited from all the participants in the RCT, including those from the control group who were compensated with the same pediatric *tuina* training [39]. The children who were included in the pilot RCT (a) were aged 4—7 years old and (b) had a score equal to or higher than the borderline cutoff of the Swanson, Nolan, and Pelham Parent Rating Scale, which indicated moderate ADHD symptoms [40–42]. The parents included in this study (a) participated in the project on using parent-administered pediatric *tuina* for ADHD symptoms in preschool children; (b) had completed the 2-month treatment and follow-up assessment of the intervention during the treatment period; and (c) agreed to participate and were willing to share their experience in applying this intervention. Parents who (a) were unable to speak Mandarin fluently and (b) had difficulties using Tencent Meeting software were excluded. Written informed consent to participate in this study was obtained from the participants.

### Data collection

The focus group was moderated in accordance with the practical guide to focus groups written by Krueger et al. [43]. A semi-structured interview guide was developed and revised on the basis of the literature review and expert comments (including two TCM practitioners and one qualitative researcher). It consisted of eight open-ended questions (Table 1). The first author (SCC) was the moderator of the focus group interviews. She had established a strong rapport with the participants because she was in charge of participant recruitment, data collection, and follow-up. The moderator was trained by a TCM practitioner with strong research expertise in TCM intervention (WFY) and an experienced qualitative researcher (HLC). In each interview session, the moderator introduced herself, explained the purpose and processes of the meeting, highlighted the rules of confidentiality, explained questions, constantly provided prompts and pauses to facilitate interactions between group members without expressing any value judgments, and ensured that the participants focused on the discussion. An assistant moderator (DDW) took notes throughout the discussion and operated recording equipment during the interviews. Participation was voluntary. No one else attended the interviews besides the participants and researchers. For confidentiality, all the participants were asked to turn off their cameras to provide a natural and safe atmosphere. The participants were reassured that although the sessions were audiotaped, no names would be recorded. Each participant was offered a gift of an ADHD-relevant book for their participation in this study.

**Table 1:** Questions for the semi-structured interview

1. Please describe your feelings on learning and applying pediatric *tuina* during the 8-week period. Prompt: the most touching or impressive experience or thought
2. Could you talk about the changes took place on you and your child after using pediatric *tuina*? Prompt: any changes (e.g. the physical functions, emotion, relationship), examples
3. If any, please talk about the difficulties you met when using pediatric *tuina*? Prompt: e.g. time, difficult manipulations
4. If any, please talk about the facilitators to motivate or help you use pediatric *tuina*. Prompt: e.g. safety of the intervention
5. If any, please talk about the barriers to insist on using pediatric *tuina* in the future. Prompt: e.g. lack of professional guidance
6. What do you think of the core content (pediatric *tuina* course and the TCM pattern identification) of the intervention? Prompt: e.g. whether useful or not
7. What do you think of the auxiliary content (pediatric *tuina* textbook, auxiliary relevant documents weekly) we provided? Prompt: e.g. whether useful or not
8. Overall speaking, do you have any suggestion for the implementation of parent-administered pediatric *tuina*? Prompt: e.g. course settings

### Data analysis

All interviews were audiotaped and transcribed verbatim in simplified Chinese prior to data analysis. Each participant was renamed by using a random generated code to ensure confidentiality. All study-related documents and transcripts were deidentified, and all audio files were destroyed once transcription was completed. Descriptive characteristics for demographic data were used to describe the sample profile. The transcripts were analyzed by using template analysis, which is a thematic analysis style that stresses the use of hierarchical coding [44, 45]. Template analysis could be roughly divided into the following steps: (a) read through the transcript for a priori themes and preliminary coding; (b) define an initial template; (c) work through all data sets systematically to modify the template by inserting, deleting, or merging contents if necessary; and (d) finalize the template for the full data set [46]. The first author (SCC) conducted the transcription and coded the data, which were subsequently reviewed for coding accuracy and consistency by another researcher (DDW). Discrepancies were resolved by consulting the principal investigator (WFY). Coding management was facilitated with MS Word software [47, 48].

### Trustworthiness

Several procedures were conducted to assure the trustworthiness of the qualitative data based on the four criteria created by Lincoln: credibility, dependability, conformability, and transferability [49, 50]. (a) Credibility: The interview proposal was revised in two group meetings, and the interview questions were pilot tested by two participants. The investigators were equipped with the required knowledge and expertise to perform their responsibilities. Two purposive sampling techniques were used. (b) Dependability: Regular debriefing sessions were held with HLC, who had sufficient experience and expertise in qualitative studies. Findings that included the code tree and quotations were provided to participants for comments and confirmation. The coding structure was validated by another researcher (DDW) and revalidated by the corresponding author (WFY) by returning to the original transcript. (c) Confirmability: The transcripts were returned to participants for feedbacks. The researchers held a critical stance and honesty through self-scrutiny to ensure that interpretations were valid and grounded in the data. Therefore, negative case analysis was adopted to enhance the trustworthiness of the qualitative data. (d) Transferability: The researchers determined the richness of the data by applying the saturation theory [51] and continued gathering data until new information had almost been exhausted by the time they reached the third session [52].

## Results

Three focus group interviews were conducted in Mandarin between November 2020 and January 2021. The interviews had the mean duration of 107 min and ranged from 77 to 127 min. Fifteen parents were invited and agreed to join. Three focus group interviews were conducted. The number of participants in each group ranged from 4 to 6. The participants did not know each other. Data saturation was applied to guide data collection [51] with themes and subthemes that were established in the first two focus group interviews and enriched after the third focus group interview.

### Sample profile

Fifteen parents (14 females [93.3%] and 1 male [6.7%]) of preschool children with ADHD symptoms, i.e., hyperactivity, anxiety, and sleep disturbance attended the three sessions. They originated from six provinces or municipalities of mainland China. The mean age of the parents was 38.2 years (SD = 5.02), and the mean age of their children was 6.33 years (SD = 0.98). The participants’ demographic information is presented in Table 2.

**Table 2:** Demographic characteristics of participants interviewed (*N* = 15)

Characteristics	All participants (*N* = 15)	Session 1 (*N* = 6)	Session 2 (*N* = 5)	Session 3 (*N* = 4)
Parents				
Age, mean (SD)	38.20 (5.02)	36.33 (4.46)	39.8 (3.35)	38.75 (7.81)
Gender, no. (%)				
Male	1 (6.67)	0 (0.00)	1 (20.00)	0 (0.00)
Female	14 (93.33)	6 (100.00)	4 (80.00)	4 (10.00)
Educational Background, no. (%)				
Secondary education	2 (13.33)	1 (16.67)	1 (20.00)	0 (0.00)
Tertiary education	13 (86.67)	5 (83.33)	4 (80.00)	4 (100.00)
Children				
Age, mean (SD)	6.33 (0.98)	5.67 (0.82)	7.00 (0.71)	6.25 (0.96)
Gender, no. (%)				
Male	14 (93.33)	6 (100.00)	4 (80.00)	4 (100.00)
Female	1 (6.67)	0 (0.00)	1 (20.00)	0 (0.00)
BMI, kg/m^2^, mean (SD)	18.98 (3.46)	19.91 (5.25)	17.69 (1.45)	19.61 (1.58)

Abbreviation: *SD* standard deviation, *BMI* body mass index, *CBT* cognitive behavioural therapy, *SIT* sensory integration therapy, *TCM* traditional Chinese medicine

### Major themes

Two themes were identified regarding the participants’ experience in applying the parent-administered pediatric *tuina* intervention and participating in the study: (1) facilitators of intervention implementation and (2) barriers to intervention implementation. The specific subthemes under each theme were described. Table 3 presents the code structure.

**Table 3:** Code structures

Themes	Sub-themes	Code Unites
Facilitators to the intervention implementation	● Perceived benefits on children and parents	● Benefits on children’s health, mainly on sleep and appetite ● Benefits on children’s emption ● Benefits on children’s learning abilities ● Benefits on parents’ moods ● Benefits on parent–child relationship
	● Acceptability of children and parents	● Parents’ acceptability (e.g., safety, convenience, feasible home setting, economic issues) ● Children’s acceptability (e.g., enjoy massage, intimacy with parents)
	● Professional supports	● TCM PI, training course, comprehensive guidance, teaching videos and books, regular information sharing
	● Parents’ expectations on long-term effects of the intervention	● Parents’ expectations on improving children’s ADHD symptoms and constitutes in a long term via the intervention
Barriers to the intervention implementation	● Limited benefits on children’s inattention symptoms	● Slow and inconspicuous effects on children’s inattention symptom
	● Manipulation management difficulties	● Parents’ uncertainties of the manipulation techniques (e.g., strength, prescription) ● Delayed onset effects of pediatric *tuina* conducted by parents
	● Limitations of TCM pattern identification	● Limitations of online TCM PI (e.g., inspection might be inaccurate; palpation was not available)

*TCM* traditional Chinese medicine, *PI* pattern identification, *ADHD* attention deficit hyperactivity disorder

#### Theme 1: Facilitators of intervention implementation

This theme consists of four subthemes: (a) perceived benefits to children and parents; (b) acceptability to parents and children; (c) professional guidance and auxiliary support; (d) and parental expectations for the long-term use of the intervention.

##### Perceived benefits to children and parents

All participants thought that their children had improved health status. They described the observable changes in the physical well-being of their children. The most prominent changes as perceived by the majority of participants were improved sleep quality and increased appetite. For example, a mother described the improvements in her child as follows: *“The main role of Tuina is reflected by the improvement in our child’s sleep. He could not sleep at 11 and 12 in the evening, but now he can even sleep in seconds in a quiet environment alone. Now he can sleep at 9 in the evening, which was previously not the case at all…He had been receiving tuina for about more than a month when I noticed that his sleep was improving…Actually, the Concerta that he received affected his appetite and sleep greatly. However, his appetite is also gradually improving after he received tuina for some time. He even eats more than ever before, which is a big surprise to me.”* (PTA-FG-14).

Many participants also mentioned changes in their children’s moods. According to their narratives, their children could control their tempers and showed patience when communicating with others. A participant mentioned: *“I used to have to repeat my question several times before he’d reply, but now he talks a lot. When he’s angry, he does not lose his temper immediately but instead tells me ‘Mom, please help me to see this and forgive me. I won’t do this again…’ His heart would melt, and he would say nice words to me. I think he lets me understand his psychological activities, and this situation is very good.”* (PTA-FG-01).

Furthermore, some participants were impressed by the improvements in their children’s academic performance and interpersonal communication in school. One participant shared: *“My biggest feeling now is that my child is making progress in his learning. He had a problem with adding and subtracting numbers beyond 10 in math, but now, although he does this slowly, he can figure it out. He did not write much before, but now he writes…. He had also learned some words from his peers. For example, he would say: ‘Mom, you were fierce to me,’ which he had before…Also, he knew fewer children before, but now he basically knows the names of his peers. He now plays games with his peers in school when he didn’t play much before… Additionally, he began to like painting and had greatly improved.”* (PTA-FG-02).

In addition to the changes in the children after the intervention, over half of the participants described their improvements in mood and mental status, citing that they “feel good” “greatly relaxed” “less anxious” and “much calmer”. *“Before I learned about ADHD, I didn't understand my boy. I felt that other children are very good, and I didn’t understand why he was always like that. I was angrier. But now, I feel that I understand him more, and he is very happy to be massaged at night. Now, when he makes a mistake, I feel that I can’t get angry because I want to know him. Therefore, my mood improved.”* (PTA-FG-05).

Some of the parents also mentioned that their relationships with children became closer. *“He probably could not feel the care and love from adults before. (Now) He slowly understood his mother’s difficulties, so now he also says, ‘Mom, let me hug you and give you a kiss.’ I am really very moved, and it is also a process of growth for myself.”* (PTA-FG-12).

##### Acceptability to parents and children

The primary advantage as felt by almost participants was that pediatric *tuina* is safe and has no or minimal side effects. Furthermore, it is an economical intervention that would not exert financial pressure on the families of children with ADHD. A mother said *“ADHD, has many treatments, but I feel that pediatric tuina is the best one. It only needs a little time, and it does not waste money. It won’t be as bad for the body as medication, and it is particularly reassuring, so I think it is particularly good.”* (PTA-FG-05).

Some participants also mentioned the convenience and accessibility of this intervention because it could be learned and delivered at home. Therefore, they did not need to go to hospitals during the COVID-19 pandemic. A parent stated that *“I used to go far to take my child to the Children’s Hospital for pediatric tuina, and I could only take them on weekends. Every time I spent 200 yuan to receive tuina only for 20 min. I was suddenly enlightened after having attended the tuina class. You have enabled me to do tuina by myself without needing to go so far.”* (PTA-FG-01).

Additionally, a few participants found that that parent-administered pediatric *tuina* was more acceptable to children than other interventions. This finding provided them a sense of security and enjoyment. *“Whenever he returned home after encountering any problem outside, he would say: ‘Mom, you please help me do tuina again, and give me a rub and a press.’ He felt that his mother’s rubbing and pressing were a kind of love, and he liked it very much…”* (PTA-FG-13).

##### Professional guidance and auxiliary support

The participants stated that the intervention was satisfactory or even went beyond their expectations. They were very impressed by the professional guidance and auxiliary support provided by the research team. Such support greatly helped them persist in practicing pediatric *tuina*. A participant expressed her satisfaction with the course sessions, timely responses from TCM practitioners, and prescriptions with manipulation videos. *“What I like is that the doctor spent a long time explaining each acupoint patiently, which is equivalent to a systematic presentation. It’s more than I expected. The teacher I contacted was also very careful, and she responded to me every time I asked anything… I took some videos and photos during the diagnosis through interrogation, and the doctor also watched the videos and photos and answered my questions*.*”* (PTA-FG-02).

The provision of corresponding textbooks and the weekly promotion of auxiliary documents met the requirements of the participants. One said that *“First, the textbooks on tuina are particularly good and the pictures are clear and in color. They completely exceeded my expectations. Second, the weekly sessions allowed us to learn and understand tuina constantly, which is also very good*.*”* (PTA-FG-03).

##### Parental expectations for the long-term use of the intervention

All parents who attended the interviews expressed their expectations regarding the effects of pediatric *tuina* on chronic symptoms (e.g., inattention), adjusting constitution, and health preservation. A mother described her hope to solve her child’s inattention problems and other aspects. *“I just hope to solve his attention problem before he turns 12, so I’m willing to anything. Furthermore, tuina can help his spleen and stomach and promote sleep, which I think are very good. Of course, we can’t see much effect in the short term, but it needs to be adhered to for the long term.”* (PTA-FG-08).

Another mother was surprised by the additional effects of pediatric *tuina* and decided to continue using it. *“I will stick to it. Originally, we did tuina for attention, but during the doctor’s lecture, we accidentally found that the spleen and stomach need to recuperate I felt that his face has changed for the better and became ruddy after about 2 weeks of recuperating, so this is also an unexpected result. Later we will persist….”* (PTA-FG-08) The parents also described their great needs for long-term professional support for their practice of pediatric *tuina*. One expressed that persistent guidance in the use of the intervention is important to her considering the safety issues in children. *“At present, I will continue to do tuina. In the future, I may stop after the doctor’s guidance is gone because still need the doctor to continuously give me some guidance to practice tuina…”* (PTA-FG-15).

Another participant mentioned her expectations of continuing to acquire knowledge in this area: *“As parents, we may still need to improve the accuracy and professionalism of our practice of tuina, and we still hope to have an opportunity to learn further the theoretical knowledge on the TCM pattern identification of tuina.”* (PTA-FG-03).

#### Barriers to intervention implementation

Barriers included the following three subthemes: (a) the limited benefits of pediatric *tuina* for children’s inattention symptoms, (b) difficulties in manipulation by parents, and (c) the limitation of online TCM pattern identification.

##### Limited benefits of pediatric tuina for children’s inattention symptoms

Despite acknowledging the physical and psychosocial benefits associated with pediatric *tuina*, more than half of the participants reported that they observed little improvements in their children’s inattention symptoms. Two participants mentioned that their children’s attention had slightly improved. Such change was usually acceptable for parents because they believed that neurological aspects require a long time to improve. A mother mentioned that *“For the moment, I don’t think that the improvement in attention is too obvious. After all, it is something in the neurological aspect and may not be obvious in the short term. Long-term persistence is needed.”* (PTA-FG-14).

##### Difficulties in manipulation by parents

The majority of the participants spoke of encountering different issues during the intervention. The major issues were the uncertainties regarding acupoint selection and manipulation strength. However, the participants actively solved the problem of acupoint selection by turning to manipulation videos and books, communicating with the TCM practitioner, or checking information on the Internet. *“The first problem is the accuracy of the acupoints, and the second problem is whether we’re using the appropriate strength. I basically solved these problems by taking a video of my own tuina practice, then taking (a video) at each acupoint and sending them to the teacher for further consultation.”* (PTA-FG-03).

Some of the participants viewed pediatric *tuina* conducted by themselves as less efficient than that conducted by TCM practitioners. A participant expressed this perception as follows: *“I don't know whether it’s my problem of strength or other problems, but I feel that the effect will be a little slower than what the doctor is saying…”* (PTA-FG-01).

Several parents were unsure whether the prescription should be adjusted in time in accordance with the changes in several factors (e.g. the season and children’s signs and symptoms). *“I still have a concern, that is, the adjustment of the acupoints. For example, his coated tongue will change again through tuina, or his liver qi is very high, and we always want to relieve his liver qi. Now, do the acupoints need to be adjusted again? Do you need to change them with the four seasons? Will the pressing of the same acupoints become useless after a long time, the way that drug resistance develops after a long time?”* (PTA-FG-13).

##### Limitation of online tcm pattern identification

The participants identified several problems, which mainly focused on TCM pattern identification, given that they implemented the intervention via online methods. A parent expressed her concerns about the accuracy of online TCM pattern identification. *“As far as I am concerned, does the TCM pattern identification need to be more detailed? How can you be sure if you do not see the child face-to-face? I have nothing to worry about except that the accuracy of TCM pattern identification is a little low.”* (PTA-FG-06).

Another parent doubted the comprehensiveness of TCM pattern identification: *“Traditional Chinese medicine still has four ways of diagnosis: looking, listening, questioning, and feeling the pulse. Pulse feeling is needed for children…. This (approach) may not be comprehensive when only the coated tongue is examined, and faces are not examined.* (PTA-FG-04).

## Discussion

This work is the first qualitative study to explore the facilitators and barriers in implementing parent-administered pediatric *tuina*. Qualitative data might enable the exploration of remarkable points that need to be examined quantitatively in further studies [53] and suggested that pediatric *tuina* has perceived benefits for the sleep quality and appetite of children and parent–child relationships but had little effect on improving children’s attention. The implementation of the intervention was feasible and acceptable for most of the parents and resulted in good adherence. The parents were satisfied with the professional support that they received and expected to receive long-term guidance and support. However, one component of the intervention, specifically, online TCM consultation was perceived to have limited accuracy, thereby leading to doubts about the accuracy of TCM pattern identification. This doubt was regarded as a major barrier.

The potential participant-centered improvements in the appetite and sleep quality of children found in this work were in line with the quantitative findings of previous clinical studies. Several clinical trials suggested the beneficial effects of pediatric *tuina* on children’s sleep quality in other pediatric conditions, such as adenoid hypertrophy [54], tourette syndrome [55], fever [56], and health maintenance [57], but no study on ADHD preschool children. A meta-analysis of 16 RCTs on the effects of baby massage for sleep quality showed that pediatric massage improved the sleep quality of infants (SMD = 0.70; 95% CI =  − 0.05 to 1.46; *p* = 0.07) [58]. In addition, the effects of pediatric *tuina* on children’s appetite was supported by several studies. A meta-analysis on pediatric massage for anorexia in children showed that massage was significantly better than medication regarding clinical effective rate (RR = 1.31, 95% CI = 1.24–1.38) [59]. Another meta-analysis suggested that pediatric *tuina* was superior to medication improving the food intake [13]. However, studies on improving sleep and appetite in ADHD children are lacking. Our finding suggested parent–child relationship has been improved during the pediatric *tuina* intervention. The effects on parent–child relationships might be related to parent training and administration patterns. A systematic review of five RCTs on parent training interventions for ADHD in children suggested that parent training might have beneficial effects on the behavior of children, reduce parental stress, and enhance parental confidence [60]. The improvements in parents and children might benefit the relationship between parents and children. Although these trainings were mostly on behavioral interventions, parent-delivered pediatric *tuina* increased family companionship, which is closely related to the progression of ADHD in children [61].

We conducted online focus interviews due to the outbreak of COVID-19. During this period, our pilot RCT was modified to be conducted online. Thus, the 64 parents who participated originated from different areas of mainland China (36 cities), and the sample of the focus group interviews was selected from these 64 parents. This method made data collection easy and safe for the parents and research team. Although online training programs for the parents of children with ADHD have been tested previously [62], this work is the first online clinical trial on pediatric *tuina* intervention. Some barriers emerged during the study process. They included the inaccuracy of TCM pattern identification due to the online diagnosis mode [63]. The diagnosis of pediatric *tuina* mainly uses inspection, listening/smelling, and inquiring instead of the four diagnostic methods of TCM for adults [64]. Among these diagnostic methods, inspection was usually limited due to online settings. Although we had taken several actions to improve the process of diagnosis (e.g., asking the participants to turn off the filter function of their video devices, sharing actual images of coated tongues taken by the TCM practitioner in advance, filling in forms with detailed conditions of their children, and booking multiple time slots to meet the TCM practitioner), some parents still have doubts about the intervention. The online study design met the demands of most participants after refinement. Given that image and audio quality vary among participants, we strongly suggest that researchers or therapists avoid completely depending on diagnoses made via video calls. The limits of online diagnosis were consistent with other evidence for telehealth supportive interventions [64–66]. Further studies may compare the effects and accuracy of online and face-to-face diagnosis in pediatric *tuina*.

Although the online implementation mode had several limitations, most parents agreed on its feasibility, convenience, and safety during the special period of the interviews. Moreover, this mode facilitated monitoring the participants. The advantages of web-based intervention were confirmed by several previous studies [64, 67, 68]. The provision of professional support and guidance for pediatric *tuina* by the research team during the whole intervention period was praised by all the participants and was undoubtedly one of the most important facilitators. Online implementation enabled instant responding to participants’ queries and providing timely professional support. It also guaranteed that regular useful information could reach the participants on time. Therefore, a combination of online professional support and face-to-face TCM pattern identification might improve the effects of pediatric *tuina*. This combination must be explored in future studies. The online parent-administered pediatric *tuina* intervention presented here could be a feasible way to provide complementary and alternative intervention to children when drugs and necessary medical equipment cannot be accessed in certain situations, such as the COVID-19 pandemic, during which many online-based interventions were developed [69–71].

Some factors, such as the parents’ expectations to improve their children’s health conditions or inattention symptoms, could be regarded as facilitators. However, the parents’ expectation to help their children can be a motivating factor for trying any new intervention and may not be a facilitator specific to parent-administered pediatric *tuina*. Therefore, we did not consider general factors for all interventions as facilitators but instead regarded them as a point related to the parents’ expectations. Pediatric *tuina* usually has slow effects for some chronic conditions, which is acceptable to the most. Therefore, the majority of the parents who attended the interview sessions expressed their intent to persist in using the intervention for its long-term effects. Similar to all parent-administered interventions, the implementation of pediatric *tuina* is inevitably time-consuming [72]. Many participants reported time management issues (e.g., they have to tend to their own business, or they have two or more children in their families, or else their children have to do homework, attend various classes, or receive physical training). However, in this project, the parents were clearly notified of the duration of intervention implementation before they signed the informed consent, and their adherence to this intervention was satisfactory. Furthermore, the intervention was implemented for 20–30 min for every other day and is thus more reasonable other parent-involved interventions. Therefore, time management was not regarded as a barrier to the implementation of this intervention.

### Strengths

First, the diverse demographic backgrounds (e.g. age, economic level, and area of residence) of the included participants increased the generalizability of this project. Second, two purposive sampling methods (homogeneity and purposeful random sampling) were combined for sample selection. Homogeneity enabled the description of a particular subgroup in depth, reduced variation, and allowed for accurate statistical analysis. Meanwhile, purposeful random sampling increased the credibility of results by including heterogeneous participants. Third, all participants were coded and advised to turn off their cameras during the video discussion, thus, encouraging them to talk openly about their experiences, especially regarding some certain sensitive topics, in front of others.

### Limitations

Rapport was established between the moderator and participants during the intervention treatment period. Although the participants actively identified the problems of the intervention, this situation might generate positive answers from the participants and lead to bias. Second, online interviews were used, and the participants’ portraits were not shown. Although this approach protected the privacy of the participants, it might lead to missing some useful information conveyed via nonverbal ways, such as body language. Third, in general, the participants had high educational levels. Two-thirds of the participants held an undergraduate degree or beyond, and among them, five participants held postgraduate degrees or beyond. This situation might limit the generalization of the study results.

### Implications

The qualitative focus group interviews enabled understanding and exploring the experiences of parents in conducting pediatric *tuina* on their children with ADHD symptoms at home; identify the facilitators and barriers in the implementation and acquire knowledge on the parents’ expectations. Further studies should be conducted to explore specifically the effects of pediatric *tuina* on the sleep quality and appetite of children and parent–child relationships by using validated measures. Furthermore, additional information-sharing sessions on ADHD, TCM, or pediatric *tuina* could be conducted to expand professional knowledge and improve the confidence and expectations of parents. In addition, pediatric *tuina* can be combined with other interventions or games that could attract the attention of children with ADHD symptoms to enhance their cooperation. The model of parent-administered pediatric *tuina* used in this project could also be applied to other children’s medical conditions.

## Conclusions

The perceived beneficial effects on children’s sleep quality and appetite and parent–child relationships, as well as timely and professional support, mainly facilitated the implementation of parent-administered pediatric *tuina*. Slow improvements in children’s inattention symptoms and the possible inaccuracies of online diagnosis were the dominant barriers to the implementation of parent-administered pediatric *tuina*. Parents have high expectations for long-term professional support during their implementation of pediatric *tuina*. The online training of parents on performing pediatric *tuina*, especially during the Covid-19 pandemic, is feasible.

## Data Availability

The data that support the findings of this study are available from the corresponding author upon reasonable request.

## Abbreviations

ADHD: Attention deficit hyperactivity disorder
RCT: Randomized controlled trial
TCM: Traditional Chinese medicine
COREQ: Consolidated criteria for reporting qualitative research
